# High genomic differentiation and limited gene flow indicate recent cryptic speciation within the genus *Laspinema* (cyanobacteria)

**DOI:** 10.3389/fmicb.2022.977454

**Published:** 2022-09-09

**Authors:** Aleksandar Stanojković, Svatopluk Skoupý, Pavel Škaloud, Petr Dvořák

**Affiliations:** ^1^Department of Botany, Faculty of Science, Palacký University Olomouc, Olomouc, Czechia; ^2^Department of Botany, Faculty of Science, Charles University in Prague, Prague, Czechia

**Keywords:** cryptic species, cyanobacteria, gene flow, phylogenomics, recombination, sympatric speciation

## Abstract

The sympatric occurrence of closely related lineages displaying conserved morphological and ecological traits is often characteristic of free-living microbes. Gene flow, recombination, selection, and mutations govern the genetic variability between these cryptic lineages and drive their differentiation. However, sequencing conservative molecular markers (e.g., 16S rRNA) coupled with insufficient population-level sampling hindered the study of intra-species genetic diversity and speciation in cyanobacteria. We used phylogenomics and a population genomic approach to investigate the extent of local genomic diversity and the mechanisms underlying sympatric speciation of *Laspinema thermale*. We found two cryptic lineages of *Laspinema*. The lineages were highly genetically diverse, with recombination occurring more frequently within than between them. That suggests the existence of a barrier to gene flow, which further maintains divergence. Genomic regions of high population differentiation harbored genes associated with possible adaptations to high/low light conditions and stress stimuli, although with a weak diversifying selection. Overall, the diversification of *Laspinema* species might have been affected by both genomic and ecological processes.

## Introduction

Comparative and population microbial genomics increased our understanding of genome evolution and genetic diversity among bacterial species (Koonin et al., 2021). Although cyanobacteria are a morphologically highly diverse group, many genera and species lack distinct phenotypic and ecological characteristics that would aid their delineation – cryptic species and genera (Casamatta et al., 2003; Dvořák et al., 2015). Whole-genome sequencing on a population level can overcome this problem by capturing fine genetic differences between cryptic species (Dvořák et al., 2017). Traditional markers such as 16S rRNA and 16S-23S ITS region are not variable enough to capture the diversity among closely related species. Population genomic studies on cyanobacteria (e.g., *Microcystis*, Pérez-Carrascal et al., 2019; *Prochlorococcus*, Kashtan et al., 2014), human pathogens (e.g., *Lactobacillus salivarius*, Harris et al., 2017), and free-living soil bacteria (e.g., *Curtobacterium*, Chase et al., 2019) revealed that genetic differences among closely related species could actually be huge, despite not being exhibited by the phenotype.

By introducing new genes or allele variations between prokaryotic lineages, the divergence leads to genomic differentiation. However, the gene flow may still be present in sympatry (Polz et al., 2013). In addition to the gene flow, rearrangements and mutations can generate the genetic variability within closely related locally coexisting bacteria and promote their differentiation (Simmons et al., 2008; Reno et al., 2009; Barraclough et al., 2012; Shapiro et al., 2012). Wiedenbeck and Cohan (2011) noted that the evolution of bacteria is affected by ecological niches. Therefore, distinct genetic groups (species) that occupy different ecological niches arose in sympatry. However, cyanobacterial studies often have a limited number of closely related strains from the same locality. Comparing genome diversity on a population level is vital to fill the gap in understanding the mechanisms driving and maintaining the divergence in coexisting strains.

Gene flow in the form of homologous and non-homologous recombination is widespread across bacterial phyla, enabling genetic exchange within and between distinct groups (Bobay and Ochman, 2017; Sheinman et al., 2021). Although this mechanism of gene exchange frequently occurs between microbes, it is not simple to detect. Significant challenges to identifying recombination events are, for instance, overall sampling effort, the number of informative sites between sequences, and the evolutionary relationships among strains (Martin et al., 2011). Strains that are more distantly related have lower signals of recombination since recombination decays with sequence divergence (Bobay and Ochman, 2017).

Gene flow occurs regardless of evolutionary relatedness and differs among microorganisms (Bobay, 2020). The recombination has a lower impact on genetic diversity in free-living terrestrial bacteria, i.e., they are mostly clonal populations, whilst it has a higher impact on others, e.g., pathogenic (Vos and Didelot, 2009). Interestingly, recombination rates among species of the same genus can be substantially different. Such patterns were observed within *Microcystis* (Pérez-Carrascal et al., 2019) and *Prochlorococcus* (Coleman and Chisholm, 2010). Moreover, lower recombination rates and higher phylogenetic distance between strains can be responsible for maintaining cohesive genetic groups within closely related microbial lineages by affecting fine ecological differences between them. The limitations to recombination are associated with barriers like sequence divergence (Cordero et al., 2012), bacterial (in)competence (Jeltsch, 2003; Carrolo et al., 2009), selection, and ecological structuring (Shapiro and Polz, 2014); thus, enabling the species divergence. The presence of almost complete or partial (limited to some genomic segments) barriers to gene exchange in a sympatric setting led to the divergence among strains of, e.g., *Sulfolobus* (Cadillo-Quiroz et al., 2012), *Vibrio* (Shapiro et al., 2012), and *Myxococcus* (Wielgoss et al., 2016). Although Pérez-Carrascal et al. (2019) showed that gene flow and recombination might be major drivers of speciation in cyanobacteria, the impact of recombination in most of them remains largely unexplored.

Terrestrial habitats harbor a tremendous diversity of bacteria (Grundmann, 2004). In this study, we investigated *Laspinema thermale*, which was first found in thermal springs (Heidari et al., 2018) and thermal mud (Duval et al., 2020). *Laspinema* is a recently described cyanobacterial genus with the type species *L. thermale*. Currently, the genus *Laspinema* encompasses several species *L. thermale*, *L. etoshii*, *L. lumbricale*, and previously misidentified *Laspinema* species - “*Oscillatoria*” *acuminata* (Zimba et al., 2020). However, due to the undersampling of this genus, the evolutionary history of *Laspinema* species is still not entirely resolved. Here, based on morphological features and phylogenetic analyses, we will refer to our strains as “*Laspinema* sp.”

We aim to investigate patterns of genetic variability within *Laspinema* sp. lineages during speciation and the processes which affect it. We reconstruct the evolutionary history of isolated strains and other cyanobacteria. We next examine pangenome and fine-scale genomic diversity among coexisting *Laspinema* sp. strains in ongoing divergence with gene flow. Finally, we investigate genomic signatures of local selection and homologous recombination (HR) in diverging lineages.

## Materials and methods

### Sample collection, whole-genome sequencing, and assembly

Two samples (D2 and D3) were collected from different sides of a puddle with a sterile spatula and placed directly in a sterile plastic bag in September 2018 in Olomouc, Czech Republic (49.57459 N, 17.281988E). The puddle is a concave water body 5–10 cm in depth, and the samples were centimeters apart. A portion of each sample was placed in 10 ml capped tubes with liquid Zehnder medium (Staub, 1961). Part of the grown biomass was then transferred to Petri dishes on solid agar Zehnder medium (1.5%) and unialgal cultures were isolated following Hašler et al. (2012). By isolating a single filament, we obtained four clonal cultures from D2 and five from the D3 sample. Altogether we had eight *Laspinema* sp. strains and one outgroup strain, *Ancylothrix* sp. Their morphology was assessed under 1,000× magnification using ZEISS Primo Star (Oberkochen, Germany) light microscope. Strains were identified following taxonomic system *sensu* Komárek and Anagnostidis (2005). The strains are maintained in culture collection at the Department of Botany, Palacký University in Olomouc. All cultures were grown in 10 ml capped tubes with liquid Zehnder medium, maintained at 22 ± 1°C and illuminated with an average photon flux density of 20 μmol photons m-^2^ s^−1^ under regime 12 h light/12 h dark.

To confirm that our strains belonged to *Laspinema* sp., we isolated genomic DNA, amplified and purified partial 16S rRNA and 16S-23S ITS region according to Stanojković et al. (2022). The PCR products were sequenced by the Sanger sequencing method at Macrogen Europe, Inc. (Amsterdam, the Netherlands).^1^ Sequences were identified using the BLAST nucleotide search.^2^ Each 16S rRNA and 16S-23S ITS sequence represented one clonal culture. GeneBank accession numbers of 16S rRNA and 16S-23S ITS sequences are in Supplementary Table S1A.

We used 100 mg of fresh biomass and extracted genomic DNA using UltraClean Microbial DNA Isolation Kit (MOBIO, Carlsbad, CA, United States), following the manufacturers’ recommendations. DNA quality and concentration were assessed using ethidium bromide-stained 1.5% agarose gel and NanoDrop 1,000 (Thermo Fisher Scientific, Wilmington, DE, United States), respectively. The DNA fragments’ size was assessed by Agilent 5,400 fragment analyzer system (Agilent Technologies, Santa Clara, CA, United States). Sequencing libraries were prepared using Nextera XT DNA Library Prep Kit (Illumina, San Diego, CA, United States). Samples were sequenced commercially on Illumina NovaSeq 6,000 platform (Novogene, United Kingdom) as a paired-end 2 × 150 bp layout. Nine whole-genome sequences were submitted to the NCBI database under the BioProject PRJNA849373.

Short raw reads were filtered and trimmed using Trimmomatic v0.39 (Bolger et al., 2014) with the following parameters ILLUMINACLIP:2:30:10, LEADING:3, TRAILING:3, SLIDINGWINDOW:4:15, and MINLEN:50. Genomes were assembled with SPAdes genome assembler v3.13.1 (Bankevich et al., 2012). Produced scaffolds were clustered into different bins by MaxBin v2.2.4 (Wu et al., 2016), where a single bin represented one genome. The completeness and contamination levels were assessed with CheckM v1.1.9 software (Parks et al., 2015). Annotations were performed using prokka v1.14.5 (Seemann, 2014).^3^

### Short read mapping and variant calling

Raw Illumina reads with a quality score of less than three and trimmed lengths of less than 50 bases were filtered and trimmed using Trimmomatic v0.39. Trimmed and filtered reads were mapped to the reference genome of “*Oscillatoria*” *acuminata* PCC 6304 (CP003607) using Burrows-Wheeler Alignment mem v0.7.17 (Li and Durbin, 2009). Duplicated reads were marked using Picard Toolkit v2.25.1 (Broad Institute).^4^ The mapped reads were sorted and indexed by SAMtools (Li et al., 2009). Variants for individual genomes without the outgroup were called using HaplotypeCaller with-ERC BP_RESOLUTION parameter within GATK v4.2.0.0 (McKenna et al., 2010). CombineGVCFs and GenotypeGVCFs modules in GATK were used to merge variant call format (vcf) files and perform genotype calls. Single nucleotide polymorphisms (SNPs) were filtered using hard-filtering parameters following GATK’s best practices pipeline and indels were removed. Additionally, we used VCFtools v0.1.16 (Danecek et al., 2011) to filter out sites with minimum allele frequency less than 0.1, mean depth values less than 40 and greater than 200, and genotype quality smaller than 30.

### Phylogenetic and phylogenomic analyses

The most similar 16S rRNA sequences were identified using BLAST nucleotide search, and in Supplementary Tables S1B–J are all hits with ≥95% sequence identities. Additionally, reference sequences of *Oscillatoria*, *Phormidium*, and other cyanobacteria were added. *Gloeobacter violaceus* (NR074282) was used as an outgroup. Multiple sequence alignment was performed by the Muscle algorithm v3.8.425 (Edgar, 2004) in AliView (Larsson, 2014). The maximum likelihood (ML) phylogeny was performed with IQ-TREE v1.6.1 (Nguyen et al., 2015) using the GTR + I + G substitution model. Ultrafast bootstrapping with 2000 replicates was used (Hoang et al., 2018).

We used Orthofinder v2.3.1 (Emms and Kelly, 2019) with default parameters to infer multiple sequence alignment and investigate evolutionary histories among cyanobacteria and *Laspinema* sp. strains. Altogether, we used 133 whole-genome sequences. Next, we performed ML phylogenetic reconstruction in IQ-TREE with the best model selected by ModelFinder (Kalyaanamoorthy et al., 2017) – LG + F + I + G4 and ultrafast bootstrap with 2000 replicates. A list of single-copy orthologues used for the phylogenomic inference can be found in Supplementary Table S2A.

Eight whole-genome sequences of *Laspinema* sp. and a reference genome “*Oscillatoria*” *acuminata* PCC 6304 were used to infer whole-genome phylogeny. We used *Ancylothrix* sp. (strain D3o) as the outgroup. Thus, our dataset encompassed ten whole-genome sequences. OrthoFinder with the option-T iqtree was employed to identify single-copy orthologues (Supplementary Table S2B), infer unrooted gene trees, and generate multiple sequence alignment. Species trees were built using three different approaches. First, the ML tree was inferred in IQ-TREE based on the LG + I + G4 substitution model from multiple sequence alignment. The tree topology was tested using ultrafast bootstrapping with 2000 replicates. Second, a coalescent-based analysis was performed with ASTRAL-III (Mirarab and Warnow, 2015; Zhang et al., 2018) using gene trees inferred from individual single-copy orthologue alignments. For the third approach, we used the variant call dataset with the only variable sites obtained from GATK. The vcf file was converted to fasta format with vcf2phylip script^4^ and then we used fasta file for the ML phylogeny inference in IQ-TREE. As the most appropriate model was selected TVM + F + ASC by ModelFinder within IQ-TREE, and 2,000 replicates were used.

The proportions of dissimilarities (p-distance) between 16S rRNA sequences were calculated in MEGA11 (Tamura et al., 2021). To assess the genomic similarity between nine genomes (outgroup omitted), average nucleotide identity (ANI) was calculated with FastANI v1.33.^5^ Both 16S rRNA and ANI matrices are in Supplementary Table S3. ANI matrix was visualized with ‘gplots’ v3.1.1 (Warnes et al., 2016) in R (version 4.1.2; R Core Team, 2021).

We used the software Saguaro v0.1 (Zamani et al., 2013) to investigate if genomic regions support different local phylogenetic relationships among aligned sequences of isolated *Laspinema* sp. strains. It detects boundaries between genomic segments that have different phylogenetic relationships and assigns “cacti” to them. Consensus genome sequences were created from the vcf file using VCFtools and whole-genome alignment was made. Saguaro was run with 20 iterations and it identified 21 cacti. We used the Saguaro2Phylip function to convert cacti into distance matrices suitable for phylogenetic analysis in Phylip v3.696 (Felsenstein, 2005) with the neighbor function. Boundaries between genomic segments with different cacti assigned to them were visualized with paint_chromosomes ruby script^6^ and the trees using the program FigTree v1.4.4 (Rambaut, 2012).

### Population genomic analyses

We investigated the pangenome of isolated *Laspinema* sp. strains and *“Oscillatoria” acuminata* PCC 6304 to reveal their genomic diversity. Genome annotations were used as an input to Roary v3.13.0 (Page et al., 2015) and pangenome was visualized using roary_plots python script.^7^ Genes were clustered into core and flexible or accessory (shell and cloud) genomes. Core genes are found in all nine genomes, shell genes are present in 2–8 genomes, while cloud genes are found in a single genome.

For analyses sensitive to population definitions, we define population D2 consisting of D2a, D2b, and D2c; and the D3 population with D3a, D3b, D3c, and D3d strains, following detected recombination between them and consistent monophyly of the two clades across phylogenies (see results and discussion). The outlier strain D2d was included as an outgroup for recombination analyses but was excluded from the rest of the analyses requiring population-level data.

To explore the differences in functionality of flexible (cloud) genes among strains of D2 and D3 lineages, we performed gene ontology (GO) enrichment analysis. Sequences of flexible genes were annotated with pannzer2 webserver (Törönen et al., 2018), and GO classes were predicted per strain. Enriched GO terms were searched using g:Profiler (Raudvere et al., 2019) using a diatom *Thalassiosira pseudonana* CCMP1335, due to the lack of species closely related to cyanobacteria in the database. GO terms with Benjamini-Hochberg FDR value of *p* < 0.05 were regarded as significant.

Intra-population diversity was assessed with nucleotide diversity (π). Inter-population genetic differentiation (F_ST_) and divergence (D_XY_) were estimated between *Laspinema* sp. strains (D2 and D3 lineages). We used package ‘PopGenome’ v2.7.5 (Pfeifer et al., 2014) in R (version 4.1.2) in 50 kb sliding windows with 12.5 kb step. One window yielded a high F_ST_ value with a few SNP sites and low read coverage and was excluded from the analysis (window coordinates: 2,837,501–2,887,501). The outputs were visualized using package ggplot2 v3.3.5 (Wickham, 2016) in R. To estimate the differences between lineages for nucleotide diversity, we performed Mann–Whitney U statistical test in R. To investigate the linkage disequilibrium (LD) over the whole genome, we calculated pairwise SNP correlation using plink v190 (Purcell et al., 2007) on a thinned D2 and D3 vcf files. Thinning was performed in VCFtools by keeping one SNP every 250 positions.

The genomes were scanned for HR using SplitsTree v4.18.1 (Huson and Bryant, 2006) and Gubbins v3.1.3 (Croucher et al., 2015). Phi test in SplitsTree calculates pairwise homoplasy index in genome alignment and reports phi statistics with assessed significances. Gubbins was run according to the software manual, with –first-tree-builder rapidnj and –tree-builder raxmlng parameters. The resulting phylogenetic tree and detected recombination sites were visualized with Phandango (Hadfield et al., 2018). We also calculated the ratio of recombination to mutation rate (ρ/θ) and the ratio of imported SNPs through recombination relative to substitution (*r/m*) between and within both populations.

### Genome-wide scans for positive selection

Genomic regions with an elevated F_ST_ value (loci within 0.99 percentile) and suppressed recombination were extracted to investigate the genomic regions responsible for the differentiation between populations D2 and D3. Potential gene functions were obtained from the UniProt protein database.^8^ Extracted nucleotide and protein sequences of genes were aligned with muscle algorithm, and codon alignments were made using perl script pal2nal (Suyama et al., 2006). On individual codon alignments of genes found in the regions of elevated F_ST_, we performed the McDonald-Kreitman (MK) test in DnaSP v6.12.03 (Rozas et al., 2017) and calculated d_N_/d_S_ ratios using SNAP v2.1.1 (Korber, 2000).

## Results

### Evolutionary relationships

Phylogenetic reconstruction of 16S rRNA using ML placed our isolates in the family Laspinemaceae, in the same clade as *“Oscillatoria” acuminata* PCC 6304, *L. etoshii*, *L. lumbricale*, *Phormidium pseudopriestleyi*, and *Perforafilum tunelii* (Supplementary Figure S1). According to the phylogenetic inference, all our sequences had the highest similarity to *L. thermale*. Short branches between our *Laspinema* sp. isolates revealed very little divergence among them. P-distance analysis of 16S rRNA confirmed that all *Laspinema* sp. strains had a high sequence similarity of 99.4–100% between each other and 98.8–99.1% similarity with *“Oscillatoria” acuminata* PCC 6304 (Supplementary Table S3A).

As these conservative markers often mask the real diversity among species, we sequenced genomes of our *Laspinema* strains. *De novo* assembly yielded genomes of length 6.5–7.4 Mb with 43.5–48% GC content. Genomes were 99–100% complete with <2% contamination. All genome features are presented in Supplementary Table S4. We reconstructed the phylogenetic relationships between eight *Laspinema* sp. strains and 125 cyanobacteria using multiple sequence alignment with 165,686 amino acid sites (Supplementary Figure S2). *Laspinema* sp. isolates clustered together in a monophyletic clade with *“Oscillatoria” acuminata* PCC 6304 and *Phormidium pseudopriestleyi* FRX01 as sister species (Supplementary Figure S2).

**Figure 1:**
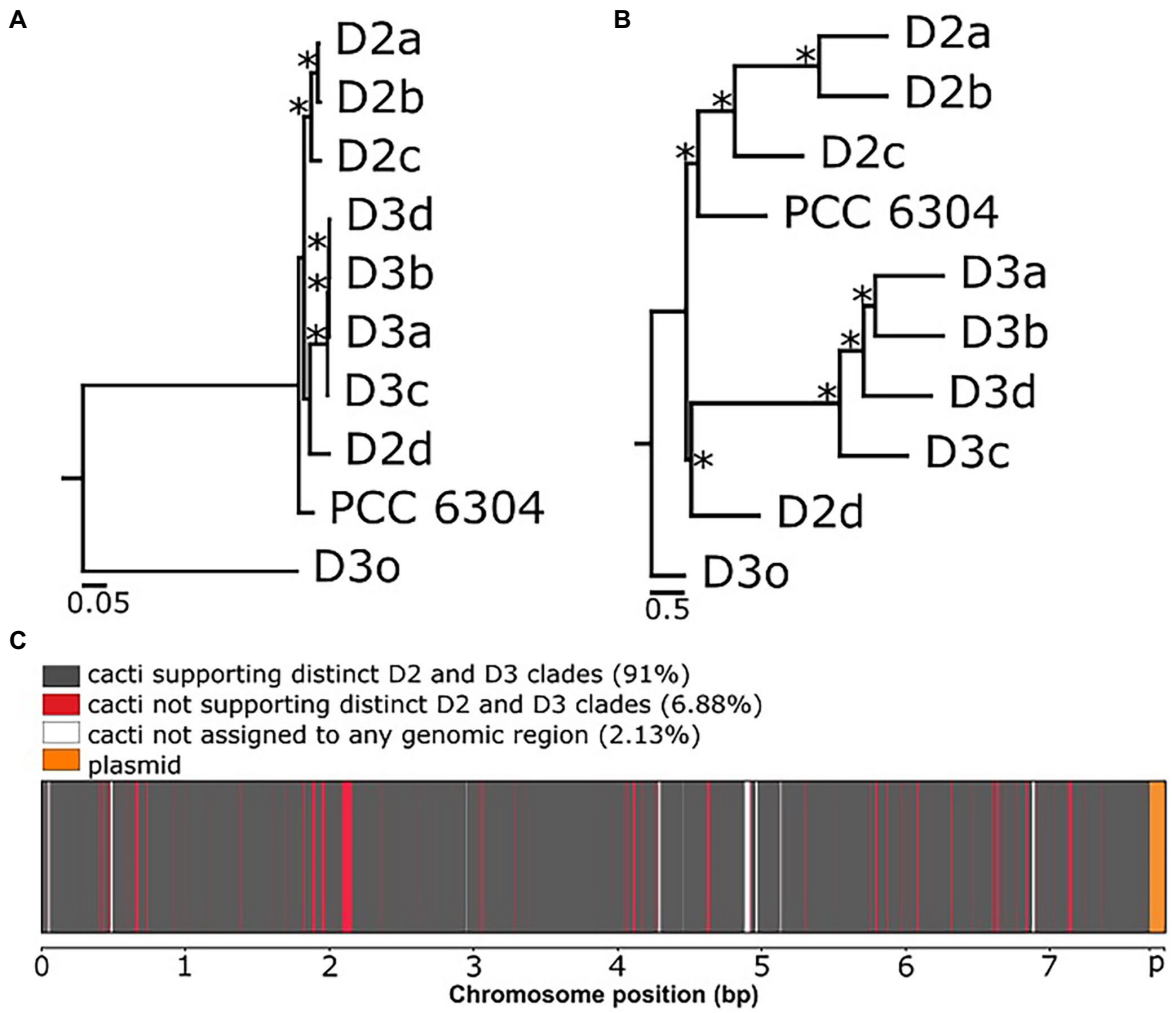
Phylogenetic relationships and genealogical discordance in the chromosome of *Laspinema*. **(A)** Maximum likelihood (ML) species tree based on 2,595 single-copy orthologues. Asterisks represent ultrafast bootstrap support ≥99. The scale bar indicates substitutions per site. **(B)** ASTRAL species tree based on individual gene trees of the 2,595 single-copy orthologues. Asterisks represent coalescent units of 1. The scale bar indicates substitutions per site. **(C)** Saguaro plot illustrating the distribution of local phylogenetic topologies over the chromosome of *Laspinema*. Genomic regions supporting the differentiation of isolates into two clades (D2 and D3) are shown in dark grey, and genomic regions not supporting it are shown in red. The letter ‘p’ marks the location of the plasmid, and it is shown in orange. Genomic regions in white were not assigned to any of the detected cacti. See Supplementary Figure S4 for further details on local topologies inferred by Saguaro with the percentage of the genome cacti covered.

Then, we reconstructed the evolutionary histories among our *Laspinema* sp. strains, the outgroup (*Ancylothrix* sp.), and *“Oscillatoria” acuminata* PCC 6304. The ML species tree was inferred from the multiple sequence alignment with 892,706 amino acid sites (Figure 1A). We then compared species trees inferred by three different approaches (aforementioned ML tree from 2,595 single-copy orthologues, ML tree from SNPs, and the ASTRAL tree from a set of unrooted gene trees under the multi-species coalescent model) to investigate the evolutionary histories among *Laspinema* sp. strains. All the analyses supported the differentiation of D3 and D2 strains in two clades, where D2d clustered with the D3 clade but with low bootstrap support of <60 (Figures 1A,B; Supplementary Figure S3). However, there was a discordance in the phylogenetic position of *“Oscillatoria” acuminata* PCC 6304. The ASTRAL analysis recovered it as a sister to the clade D2, while in the ML analysis, it appeared more distantly related, with low bootstrap support (<60).

**Figure 2:**
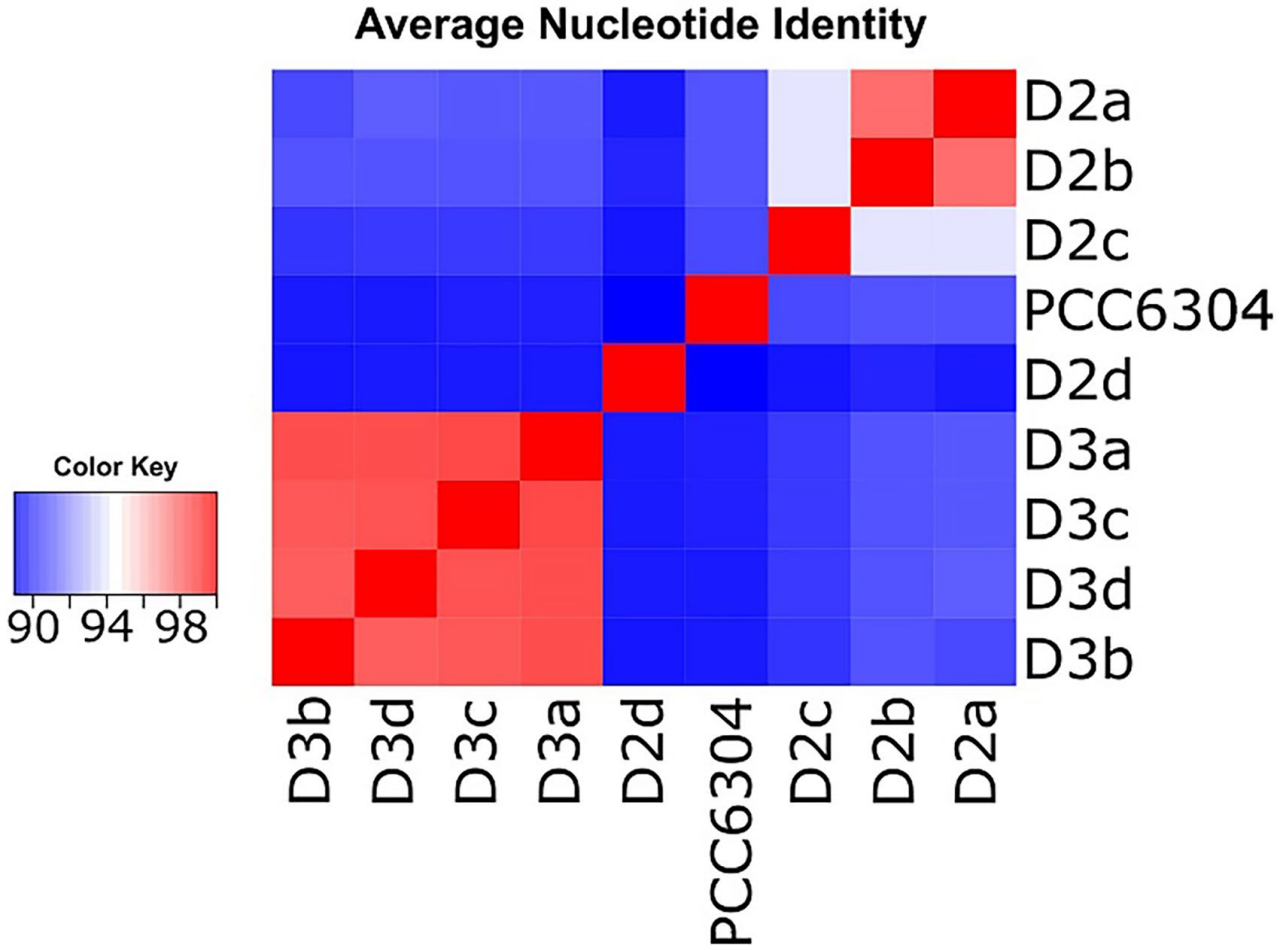
Pairwise genome-wide average nucleotide identity (ANI) heatmap showing 8 *Laspinema* sp. strains along with the reference strain *“Oscillatoria” acuminata* (marked PCC 6304). Red indicates a high ANI and blue indicates a lower ANI value. The scale bar represents color codes of ANI values, showing sequence identity percentage. See Supplementary Table S3B for the exact ANI values. The heatmap was created with heatmap.2 function within ‘gplots’ (Warnes et al., 2016) package in R.

The analyses showed that isolates D2a and D2b shared 97.6% ANI, while D3a, D3b, D3c, and D3d, shared 97.9–98.4% sequence identity across their genomes (Figure 2; Supplementary Table S3B). All *Laspinema* isolates had <91% similarity to *“Oscillatoria” acuminata* PCC 6304. Strain D2c had 93.9% genome similarity to D2a and D2b strains and <91% genome similarity to the D3 population, whereas the D2d shared the lowest sequence identity with all other strains (<90% ANI). In other words, following the 95% ANI threshold for species delineation (Goris et al., 2007), our dataset consisted of five species – D2, D3, D2d, D3c, and *“Oscillatoria” acuminata* PCC 6304.

We used the Saguaro program to infer the distribution of the phylogenetic tree topologies over the genome. The program identified 1,237 segments and 21 cacti (Figure 1C). The length of genomic segments and cacti assigned to them are in Supplementary Table S5, and the percentage distribution of every cactus is in Supplementary Figure S4. Ten detected cacti covering 91% of the genome featured the expected differentiation of isolates into two clades, D2 and D3. The rest of identified cacti supported different local topologies and covered 6.88% of the genome (Figure 1C; Supplementary Figure S4). Cacti not assigned to any region covered 2.13% of the genome. The most common cacti (cactus4–27.22% and cactus0–24.44% of the genome) were scattered across the genome, and they represented topologies featuring the expected clade differentiation (Supplementary Figure S4).

The strain D2d likely represents a separate lineage due to its unresolved phylogenetic position, low sequence identity, pangenome content, and the lack of recombination events it shared with other *Laspinema* sp. isolates. As we lacked closely related strains to the D2d, we excluded it from the rest of the analyses based on pre-defined populations.

### *Laspinema* sp. pan-genome

**Figure 3:**
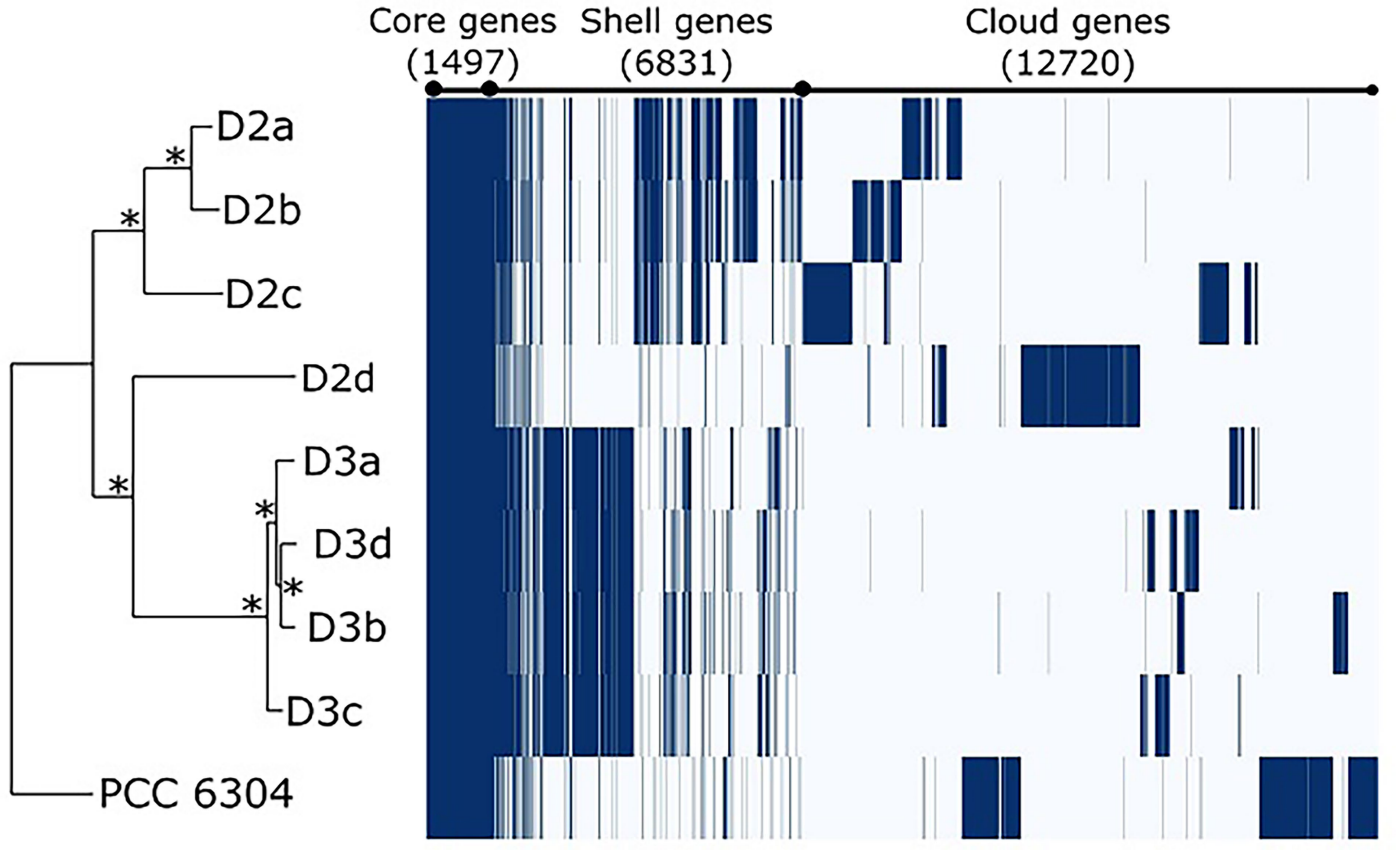
Gene presence and absence matrix from pangenome analysis of 8 *Laspinema* strains and *“Oscillatoria” acuminata* (marked PCC 6304) inferred by Roary (Page et al., 2015). Core genes represent genes present in all strains, shell genes appear in 2–8, while cloud genes are in a single strain. Each row corresponds to the branch of the phylogenetic tree, while each column represents the orthologous gene family. The dark blue line indicates the presence of a gene, while the white blocks indicate the absence of a gene. Asterisks in the ML tree indicate bootstrap values of 100.

As a result of pangenome analysis, Roary clustered all coding DNA sequences (CDS) in the core and flexible genome (shell and cloud). The core genome included genes present in all nine strains; the shell had genes present in 2–8 strains, while the cloud genome had genes present in a single strain. Altogether, *“Oscillatoria” acuminata* PCC 6304 and eight *Laspinema* sp. strains had a pangenome of 21,048 genes (Table 1; Figure 3). The core genome had 1,497 CDS–7.11% of the pangenome and the flexible (shell) had 6,831 CDS–32.45% of the pangenome (Table 1). Moreover, the flexible genome (cloud) accounted for 60.43% of the pangenome with 12,720 singleton genes. In all isolates, the core genome accounted for 26.28–29.16% of the whole genome (Supplementary Table S4).

**Table 1:** The proportion of protein-coding genes in the core, shell, and cloud of eight *Laspinema* sp. strains and “*Oscillatoria*” *acuminata* PCC6304 pangenome.

	Strain number	Gene number
Core genes	9	1,497
Shell genes	2–8	6,831
Cloud genes	1	12,720
Total genes		21,048

We then performed GO enrichment analysis on genes present only in the flexible genome (cloud) of D2 and D3 populations. Classification by molecular function showed that both populations had a number of genes in charge of catalytic activity (GO:0003824), organic cyclic compound binding (GO:0097159), and ion binding (GO:0043167). Under the catalytic activity category, there were genes responsible for oxidoreductase activity (GO:0016491), transferase activity (GO:0016772), and kinase activity (GO:0016301), etc. Classification by biological process included genes related to the metabolic processes, e.g., organic substance metabolic process (GO:0071704) and nitrogen compound metabolic process (GO:0006807). According to cellular component classification, there were genes regulating cellular anatomical entity (GO:0110165) and integral component of membrane (GO:0016021). The D3 population had GO terms associated with carbohydrate binding (GO:0030246), metal cluster binding (GO:0051540), and iron–sulfur cluster binding (GO:0051536) that were not found in the D2. Contrarily, the D2 population had more unique GO terms. They were genes responsible for different catalytic activities, e.g., ATP-dependent activity (GO:0140657) or helicase activity (GO:0004386), and metabolic processes, e.g., biosynthetic process (GO:0009058), protein metabolic process (GO:0019538), etc. A list of all GO terms, their functions, adjusted *p*-values associated with them, and highlighted differing GO terms of populations are presented in Supplementary Table S6.

### Population differentiation

**Figure 4:**
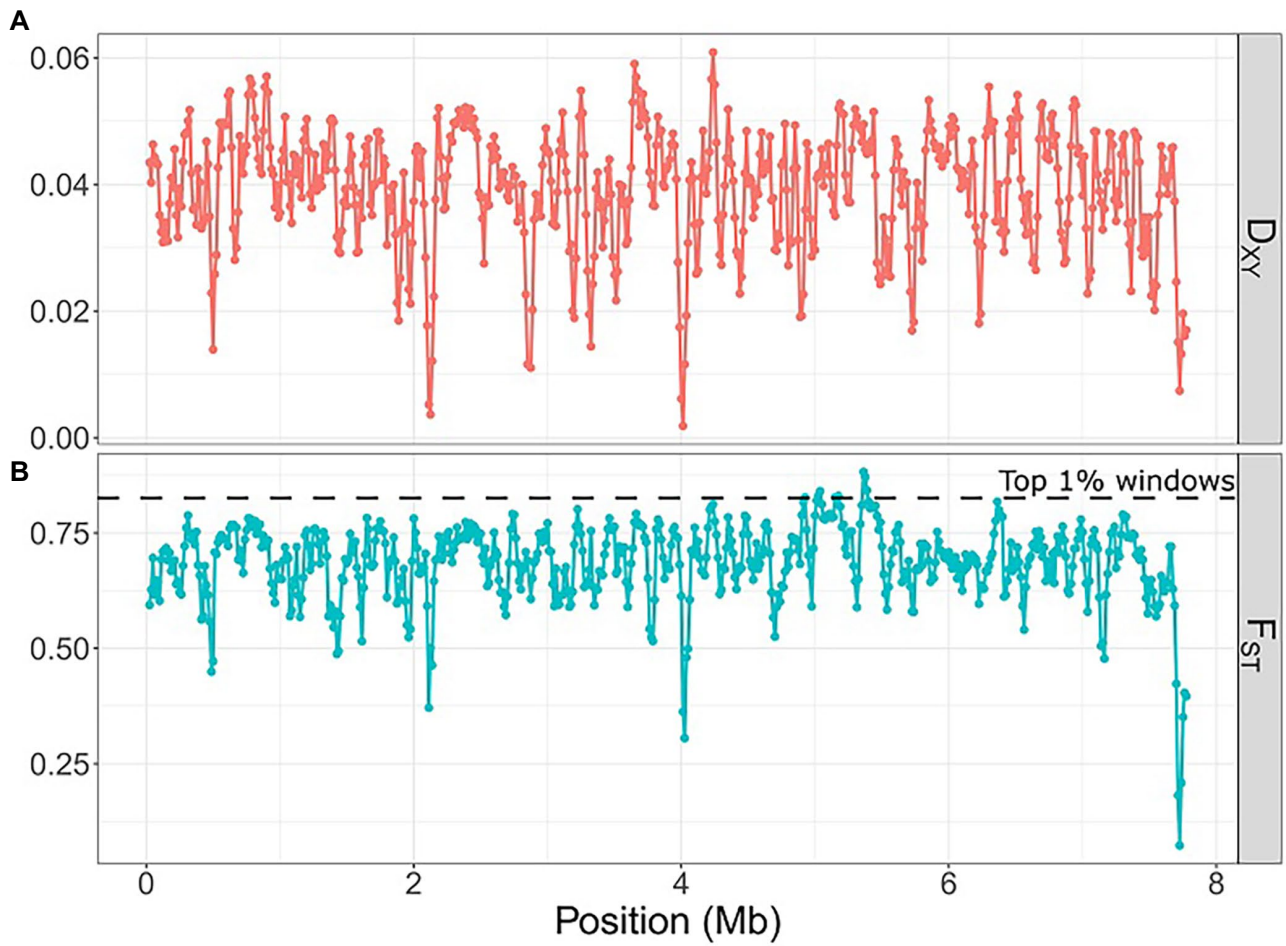
Scatter plot of absolute divergence (D_XY_) and fixation index (F_ST_) between the D2 and D3 *Laspinema* populations using 50 kb windows with 12.5 kb step along the *Laspinema* genome. The horizontal dashed line indicates loci within the 0.99 percentile of F_ST._ Scatter plots were created using the ‘ggplot2’ (Wickham, 2016) package in R. **(A)** D_XY_, **(B)** F_ST_.

We used four metrics (F_ST_, π, D_XY_, Tajima’s D) to estimate genetic diversity and population differentiation among *Laspinema* sp. isolates. The mean nucleotide diversity was 0.0179 for D2 and 0.0059 for the D3 population (Supplementary Figure S5). The nucleotide diversity of D3 was significantly lower than D2 (Mann–Whitney test, *p* < 2.2e-16). The mean absolute divergence at the interspecific level was 0.039 and the mean fixation index was 0.69 (Figure 4). In genomic regions of elevated F_ST_ value (99th percentile) and suppressed recombination, we found 26 annotated genes. Seven genes were associated with different metabolic processes, three with DNA or RNA processing, five with adaptation to low/high light conditions, and 11 with adaptation to stress stimuli (Supplementary Table S7). Most of the loci were under weak selection (Supplementary Table S8).

Then we assessed the impact of HR on *Laspinema* sp. genome diversity. Phi statistics (the pairwise homoplasy index) supported that HR was present within *Laspinema* sp. isolates (*p* < 0.0001). The mean *r*^2^ value was 0.702 for D2 and 0.413 for the D3 population indicating a medium linkage between loci over the genomes, consistent with the higher HR among D3 strains. Identified recombination fragments detected by Gubbins are presented in Supplementary Figure S6. We also calculated the ρ/θ and *r*/*m* ratios between and within D2 and D3 populations. Averaged per-branch ratios of ρ/θ and *r*/*m* were low for D2 (ρ/θ = 0.016; *r*/*m* = 0.822), whereas they were intermediate in D3 (ρ/θ = 0.023; *r*/*m* = 1.368). Moreover, low recombination rate was detected between populations (ρ/θ = 0.011; *r*/*m* = 0.608). A summary of *r*/*m* values between different prokaryotic species is shown in Table 2. The fraction of the genome affected by HR was between 3.27–12.98% for the D2 and 9.05–11.67% for the D3 population. The mean fraction of the genome subjected to HR was 10.06% (Supplementary Table S9). Gubbins detected almost no HR of D2d with other *Laspinema* sp. strains, with 0.23% of its genome being subject to HR.

**Table 2:** The relative effect of recombination and mutation (*r*/*m*) for different microbial species based on whole genomes or multilocus sequence analysis from previous studies.

Species	Phylum	*r/m*	Reference
*Laspinema* sp. (D3)	Cyanobacteria	1.368	This study
*Laspinema* sp. (D2)	Cyanobacteria	0.822	This study
*Laspinema* sp. (D3&D2)	Cyanobacteria	0.608	This study
*Streptococcus pneumoniae*	Firmicutes	0.0–45.4	Chaguza et al. (2016)
*Listeria monocytogenes*	Firmicutes	0.01–0.5	Zamudio et al. (2020)
*Vibrio parahaemolyticus*	γ-proteobacteria	1.5–24.3	Martinez-Urtaza et al. (2017)
Microcystis aeruginosa*	Cyanobacteria	1.5–13.9	Pérez-Carrascal et al. (2019)
Sulfolobus islandicus*	Thermoprotei (Archaea)	3.8	Held et al. (2010)

Asterisks mark *r*/*m* ratios obtained with the program other than Gubbins.

## Discussion

We used the population genomic approach to gain deeper insight into the evolutionary processes of closely related free-living cyanobacteria within the genus *Laspinema*. We found two new cyanobacterial lineages that are highly genetically divergent, despite having almost identical 16S rRNA sequences and being isolated from the same locality. The pangenome analysis revealed that gene content within the flexible genome differs between them and might be responsible for the local adaptation. Moreover, the lineages exhibit low recombination rates between them. Finally, the two lineages had suppressed recombination in genes associated with the adaptation to low/high light conditions and stress stimuli, suggesting their tentative role as niche-adaptive genes. Our results indicate that the two lineages are in the process of divergence, which might be driven by both ecological and genetic separation.

### Thin line between cryptic lineages and species within *Laspinema thermale*

Many microbial taxa consist of morphologically almost indistinguishable but genetically differentiated lineages. These cryptic lineages are widespread across prokaryotes imposing significant issues on their taxonomic resolution (Dvořák et al., 2014; Walk, 2015). This issue arises due to the inconsistent application of species concepts, low phenotypic diversity among strains, and the limited sampling effort of many prokaryotic taxa (Ward et al., 2008). Some authors had already employed ecological and genetic data to overcome cryptic diversity and describe morphologically similar or identical closely related cyanobacteria (e.g., *Symplocastrum*, Pietrasiak et al., 2014). Moreover, screening the flexible genome for intrapopulation gene content and function variation could reveal if distinct lineages are associated with distinct ecological niches (Shapiro and Polz, 2014). However, detecting such differences in gene content is hindered by the presence of many genes with still unknown functions (Rodriguez-Valera and Ussery, 2012). Other authors suspected that lineages might represent different species if they formed distinct genetic clusters occupying different ecological niches with limited gene flow between them (*Vibrio*; Hunt et al., 2008; *Sulfolobus*; Cadillo-Quiroz et al., 2012; *Microcystis*; Pérez-Carrascal et al., 2019). We observed similar patterns of lineage differentiation in *Laspinema*. We suspect the divergence of sympatric D2 and D3 lineages was initiated by ecological heterogeneity, and the prevalence of population-specific recombination maintains them as distinct genetic clusters. That would be consistent with the biological species concept and dominant gene flow within rather than between lineages (Mayr, 1942; Shapiro and Polz, 2015). Both lineages have low morphological diversity (data not shown) and almost complete barriers to gene flow (discussed below), but they are still in the intermediate, so-called “grey zone” of speciation (Roux et al., 2016; Kollár et al., 2022). The D2 and D3 are on separate evolutionary pathways, likely to further diverge in time and space.

Nevertheless, additional investigations of the exact ecological and morphological differences as well as (dis)continuity of gene flow between the cryptic species are required to characterize them fully.

### Whole-genome sequencing facilitates the taxonomic resolution of cyanobacteria

Reconstructing the evolutionary histories and understanding the extent of cyanobacterial diversity are hindered by insufficient population-level sampling (Pietrasiak et al., 2019). The sequencing of conservative molecular markers has been a cornerstone for the characterization of prokaryotic organisms. Hence, a much larger genetic diversity among closely related microbes has been overlooked (Jaspers and Overmann, 2004; Yarza et al., 2014). Here, the ML phylogeny of 16S rRNA and p-distance similarity matrix (16S rRNA) showed that all *Laspinema* sp. isolates belong to one species – *L. thermale* (Supplementary Figure S1; Supplementary Table S3A). The strains exhibited short branches and clustered together, which suggests a low level of divergence among them (<0.55% divergence in 16S rRNA).

Nevertheless, employing whole-genome sequencing of closely related coexisting strains allowed us to detect the separation of *Laspinema* sp. into two highly supported (bootstrap value 100) well-differentiated subclades – D2 and D3 (Figures 1A,B; Supplementary Figures S3, S4). The discordant position of *“Oscillatoria” acuminata* PCC 6304 in the species trees indicates a potential presence of the gene tree conflict due to the incomplete lineage sorting (Figure 1B). Besides, the strain D2d had an unresolved position in the species tree with low support (Figure 1A). These results support the existence of at least four young species within *Laspinema* and highlight that more extensive population-level sampling is required to resolve phylogenetic relationships between them.

Previous studies suggested a 95–96% ANI cutoff for species delineation (e.g., Goris et al., 2007; Yarza et al., 2014). According to the 95% threshold, the ANI analysis yielded five independent lineages – D2, D3, *“Oscillatoria” acuminata* PCC 6304, D2c, and D2d (Figure 2). However, the characterization of new species based on arbitrary sequence thresholds can be ambiguous as they disregard evolutionary causes of divergence (see Bobay, 2020). For instance, mutation rates, genetic exchange, and selection pressures can significantly differ among microbes (Vos and Didelot, 2009; Bobay, 2020). Thus, the 95% ANI threshold for species delineation is likely to be lower in *Laspinema,* and we consider D2c to be a part of the lineage – D2, which was consistently monophyletic with the D2a and D2b strains in phylogenetic analyses. As previously highlighted, we suspect the D2d strain might be a new species. Still, due to the lack of population data, we excluded it from analyses that needed pre-defined populations.

Conflicts in phylogenetic relationships among organisms arise from incongruent evolutionary histories between individual genes or different parts of the genome (Galtier and Daubin, 2008). The extent of phylogenetic incongruence in microbes has rarely been researched genome-wide (including coding and non-coding regions), especially in cyanobacteria. We used Saguaro to detect and visualize conflicting topologies over the whole chromosome (Figure 1C; Supplementary Figure S4). Although ML and ASTRAL species trees revealed high support for the phylogenetic relationships between our *Laspinema* strains, we noted that 6.88% of the genome is discordant with the species tree topology. Those regions do not support distinct monophyletic clades of D2 and D3, and they could be affected by incomplete lineage sorting or gene flow (Galtier and Daubin, 2008). Genomic segments supporting the differentiation of *Laspinema* sp. isolates into two monophyletic clades comprise 91% of the genome (10 cacti; Figure 1C; Supplementary Figure S4) and indicate that they are likely separate species. A similar example of scattered congruent and incongruent topologies across the genome has been found in animals – *Heliconius* butterflies (Martin et al., 2013).

Sympatric, cryptic Laspinema lineages D2 and D3 are substantially genetically differentiated (Figures 2, 3), but they lack distinct morphological characters (data not shown). Whole-genome sequencing facilitated the taxonomic resolution of cyanobacteria and we demonstrated the existence of two likely new species within *L. thermale*, despite cryptic diversity.

### Extensive diversity of *Laspinema* sp. pangenome

The pangenome analyses revealed intraspecies genetic diversity and further supported that strains D2d and “*Oscillatoria*” *acuminata* PCC 6304 as well as clades D2 and D3 represent co-occurring genetically distinct lineages in *Laspinema* sp. Each lineage had its own block of genes within the flexible genome (cloud+shell), i.e., the gene pool of *Laspinema* sp. is extensive (Figure 3). These gene blocks accounted for ~60% of the whole genome, a common proportion observed in free-living microbes (McInerney et al., 2017).

The pangenome compositions differed between the D2 and D3, which is explained by strains sharing more similar blocks of flexible genes within respective lineages (Figure 3). Given that their flexible genome (cloud) was enriched with genes involved in respiratory chains, regulation of responses to the environment as well as transport of substrates and their metabolic processing, it is likely that one fraction of them might be advantageous and involved in niche adaptation (Rodriguez-Valera and Ussery, 2012; Shapiro et al., 2012). Moreover, the functional differences of flexible genes may contribute to the better substrate utilization of one lineage over another (Supplementary Table S6). For instance, the D3 lineage may be better adapted for the uptake of carbohydrates and limiting micronutrients (e.g., iron), and the D2 may synthesize organic compounds that would improve its response to various environmental stimuli (Rodriguez-Valera et al., 2016). However, there is still a high percentage of genes with unknown functions in the *Laspinema* pangenome (76.9%), which might have arisen due to the ecological divergence between the lineages.

Our study aligns with previous observations that conservative markers are insufficient to capture the true extent of microbial intraspecies diversity (Richter and Rosselló-Móra, 2009; Zhaxybayeva et al., 2009). Moreover, applying various population genomics approaches to characterize new species offers a better understanding of the fine microbial diversity than using different thresholds of sequence similarities.

### High genomic divergence and recombination suppression of light and stress adaptation genes in D2 and D3

Genome-wide F_ST_ and absolute divergence (D_XY_) that exceeded nucleotide divergence both suggest high genetic differentiation between D2 and D3 lineages. The majority of the genome exhibits high values of F_ST_ (mean F_ST_ of 0.69; Figure 4), which could indicate the presence of diversifying selection among many loci scattered across the genome. Additionally, it highlights low gene transfer between the lineages. We investigated potential signatures of the selection in genomic segments with a high level of divergence (99th percentile of F_ST_ values), low nucleotide diversity, and suppressed recombination. Tentative adaptive genes that were localized in these regions regulate physiological processes associated with the exposure to high/low light conditions, sugar and protein metabolism, and the response to various environmental stresses, e.g., nitrogen, phosphate, or magnesium deprivation (Supplementary Table S7). Experimental and genomic studies of closely related marine cyanobacteria *Prochlorococcus* and *Synechococcus* identified some genes that could be associated with adaptations to ecological niches (Rocap et al., 2003; Stuart et al., 2013; Kashtan et al., 2014). Detected genes were involved in, e.g., nutrient uptake (nitrogen, phosphorus, iron), light acclimation, regulatory functions, and response to various environmental stimuli. Similar patterns were discovered in other microbes like *Vibrio* (e.g., stress-response genes; Shapiro et al., 2012) and *Curtobacterium* (carbohydrases; Chase et al., 2019).

We detected a weak signal of diversifying selection on potential adaptive genes in *Laspinema* using the MK test (Supplementary Table S8), which suggests that loci evolve neutrally. Consistent with observations in *Sulfolobus* (Cadillo-Quiroz et al., 2012), maintenance of multiple adaptive alleles might be beneficial for the coexistence of *Laspinema* lineages and the introduction of small differences in their fitness. These fine differences are linked to a wide range of niches that can be really small in soil ecosystems – microniches (Vos et al., 2013). *L. thermale* is well-adapted to diverse soil habitats - from radioactive thermal springs (Heidari et al., 2018) with extreme temperatures and contamination to puddles, which frequently undergo substantial environmental changes (e.g., drying, wetting, nutrient limitation, exposure to UV light). Genes regulating phototaxis (cheW, CheY, cheB) and response to various environmental stimuli (rcsC, rssB) exhibited a strong interspecific structuring, i.e., they were different genes associated with the same function (Supplementary Table S8). This could indicate the separation of ecological niches between D2 and D3, which might have initiated the process of ecological differentiation in *Laspinema* lineages (Shapiro et al., 2012). Hence, one lineage may grow better in unfavorable conditions or burrow deeper in the soil due to the excess light or lack of moisture (Mager and Thomas, 2011). Nevertheless, the functions of niche-specific genes require future physiological investigations to confirm whether these genes are directly involved in adaptive processes.

### Recent speciation of cryptic *Laspinema* sp. lineages

Many microbial species are in different stages of speciation, which has been proposed to be driven by the adaptation to various ecological niches (Polz et al., 2013; Shapiro and Polz, 2015). *Laspinema* exhibits the overall genomic pattern of high genetic divergence and neutral evolution in highly differentiated genomic regions. Besides, a trend of decreased HR and high LD suggests the clonal nature of our *Laspinema* strains. The observed recombination rates concur with previous estimates for some cyanobacteria (e.g., *Microcoleus*) and free-living prokaryotes (Vos and Didelot, 2009; González-Torres et al., 2019), suggesting that *Laspinema* strains engage in little recombination compared to mutation (Table 2). Following the *r*/*m* ratio, which is the relative impact of recombination and mutation on lineage diversification, population-specific recombination is stronger in the D3 (*r*/*m* = 1.368) than in the D2 lineage (*r*/*m* = 0.822). In spite of occurring less frequently than mutation (D3_ρ/θ_ = 0.023), recombination events introduced almost 1.5 as many substitutions as mutations. That highlights the importance of HR over mutation in shaping the genetic diversity of the D3 lineage. However, lower recombination rates among D2 strains could be an artifact of fewer individuals in that clade. Such a pattern of reduced gene flow between lineages (*r*/*m* = 0.608) indicates the existence of a recombination barrier which may contribute to the maintenance of strains in cohesive genetic groups and a further spread of tentative adaptive genes for niche specialization (Fraser et al., 2007).

Given all of the patterns of genetic differentiation among D2 and D3, following Shapiro and Polz's (2015) bacterial speciation model, we estimate that *Laspinema* lineages might be at speciation stage 3 or 4. Although we detected weak diversifying selection in tentative adaptive genes, established genomic isolation with reduced gene flow between lineages could suggest that *Laspinema* is in ongoing ecological differentiation, which may reach completion.

Notably, a low number of individuals in both lineages might have impacted our observations. Thus, we avoided analyses requiring larger population sampling and pre-defined populations. Another limitation is the lack of population genetic data for isolated strain D2d, which is likely a separate species. However, due to the random sampling and the high diversity of terrestrial microbial communities, finding strains closely related to that specific one would be extremely challenging. Forthcoming physiological and modeling studies are crucial for testing the adaptive differences between two lineages and discovering genes of unknown function, which harbor significant evidence for unexplored ecological niches in soil.

Overall, we provide a deeper insight into the genetic diversity that underlies the divergence of cyanobacterium *Laspinema*. We have highlighted the importance of using the population genetics approach over traditional markers in studying cyanobacterial diversity. We identified two cryptic genetically differentiated *Laspinema* lineages that could represent new cyanobacterial species. A suite of potential adaptive alleles associated with specialization to niches in the soil already emerged in the two lineages coexisting in a sympatric setting, although with a weak signature of diversifying selection. Lineage divergence might be driven by genomic and ecological processes and is further maintained by the limited gene flow between them. The origin of barriers to gene flow among *Laspinema* strains remains to be investigated.

## Data availability statement

All 16S rRNA and 16S–23S ITS sequences have been deposited in GenBank under accession numbers ON814838-ON814846 and ON814847-ON814854, respectively. Biosample identification and accession numbers are available in Table S4. Relevant codes used in the analyses are available at https://github.com/AleksandarStan/Laspinema. The vcf file and multiple sequence alignments (for whole-genome and 16S rRNA phylogeny and extracted from the vcf file) can be found on figshare (https://doi.org/10.6084/m9.figshare.20116118).

## Author contributions

AS, SS, and PD designed the research and analyzed the data. AS wrote the paper and conducted the research. All authors contributed to the article and approved the submitted version.

## Funding

This research was funded by the Internal Grant Agency of Palacký University (grant no. IGA-PrF-2022-002) and the Grant Agency of the Czech Republic (grant no. 19-12994Y).

## Conflict of interest

The authors declare that the research was conducted in the absence of any commercial or financial relationships that could be construed as a potential conflict of interest.

## Publisher’s note

All claims expressed in this article are solely those of the authors and do not necessarily represent those of their affiliated organizations, or those of the publisher, the editors and the reviewers. Any product that may be evaluated in this article, or claim that may be made by its manufacturer, is not guaranteed or endorsed by the publisher.

## Supplementary material

The Supplementary material for this article can be found online at: https://www.frontiersin.org/articles/10.3389/fmicb.2022.977454/full#supplementary-material

## Footnotes

^1^ http://dna.macrogen-europe.com

^2^ http://blast.ncbi.nlm.nih.gov/Blast.cgi

^3^ https://github.com/tseemann/prokka

^4^ https://github.com/broadinstitute/picard

^4^ https://github.com/edgardomortiz/vcf2phylip

^5^ https://github.com/ParBLiSS/FastANI

^6^ https://github.com/IsmailM/

^7^ https://github.com/sanger-pathogens/Roary/

^8^ https://www.uniprot.org
