# Supplementary material for: High genomic differentiation and limited gene flow indicate recent cryptic speciation within the genus Laspinema (cyanobacteria)
Source: Front Microbiol. 2022 Sep 9;13:977454. doi: 10.3389/fmicb.2022.977454 (PMC9500459; doi:10.3389/fmicb.2022.977454)
Supplement: Supplementary file 1 [file Data_Sheet_1.ZIP › Supplementary Figure S1.pdf]

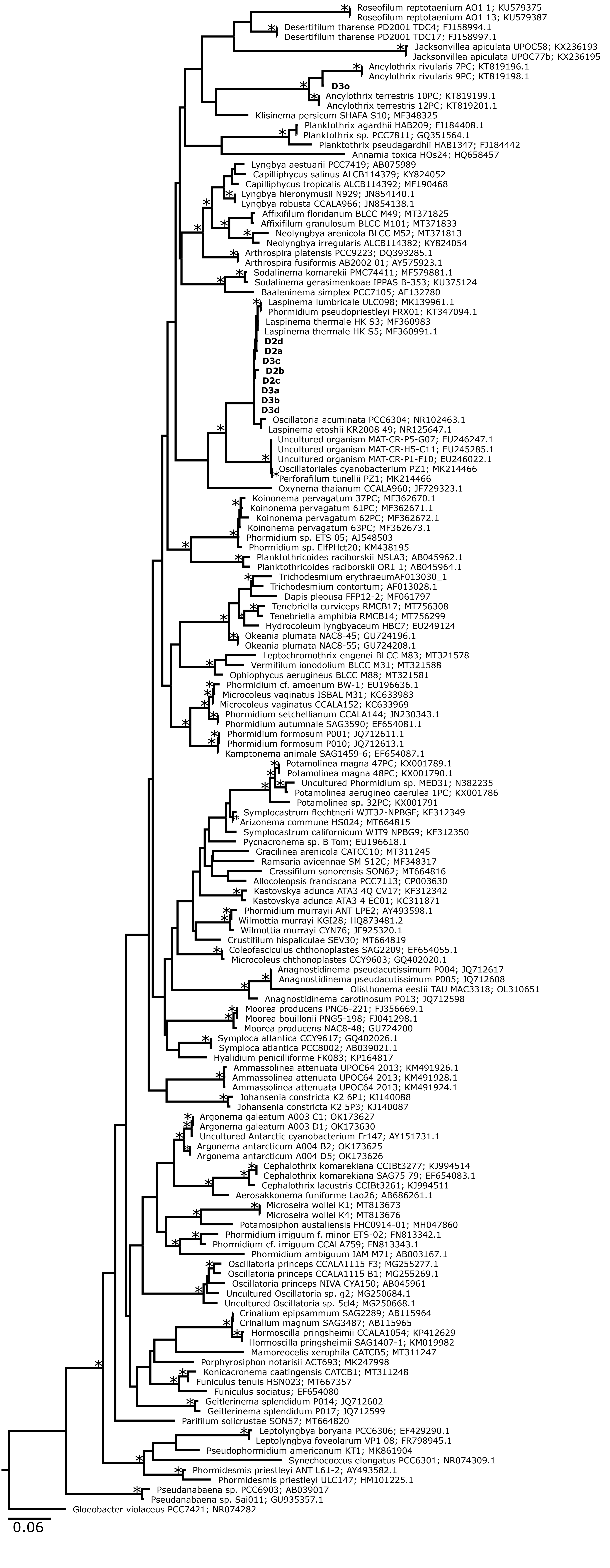

Supplementary Figure S1. Phylogenetic tree inferred from the maximum likelihood (ML) analysis based on 154 16S rRNA sequences of our *Laspinema* and *Ancylothrix* strains (in bold) as well as other cyanobacteria. Asterisks at the nodes indicate maximum likelihood bootstrap support of 99 or 100. The scale bar indicates substitutions per site.
