# Supplementary material for: High genomic differentiation and limited gene flow indicate recent cryptic speciation within the genus Laspinema (cyanobacteria)
Source: Front Microbiol. 2022 Sep 9;13:977454. doi: 10.3389/fmicb.2022.977454 (PMC9500459; doi:10.3389/fmicb.2022.977454)
Supplement: Supplementary file 1 [file Data_Sheet_1.ZIP › Supplementary Figure S3.pdf]

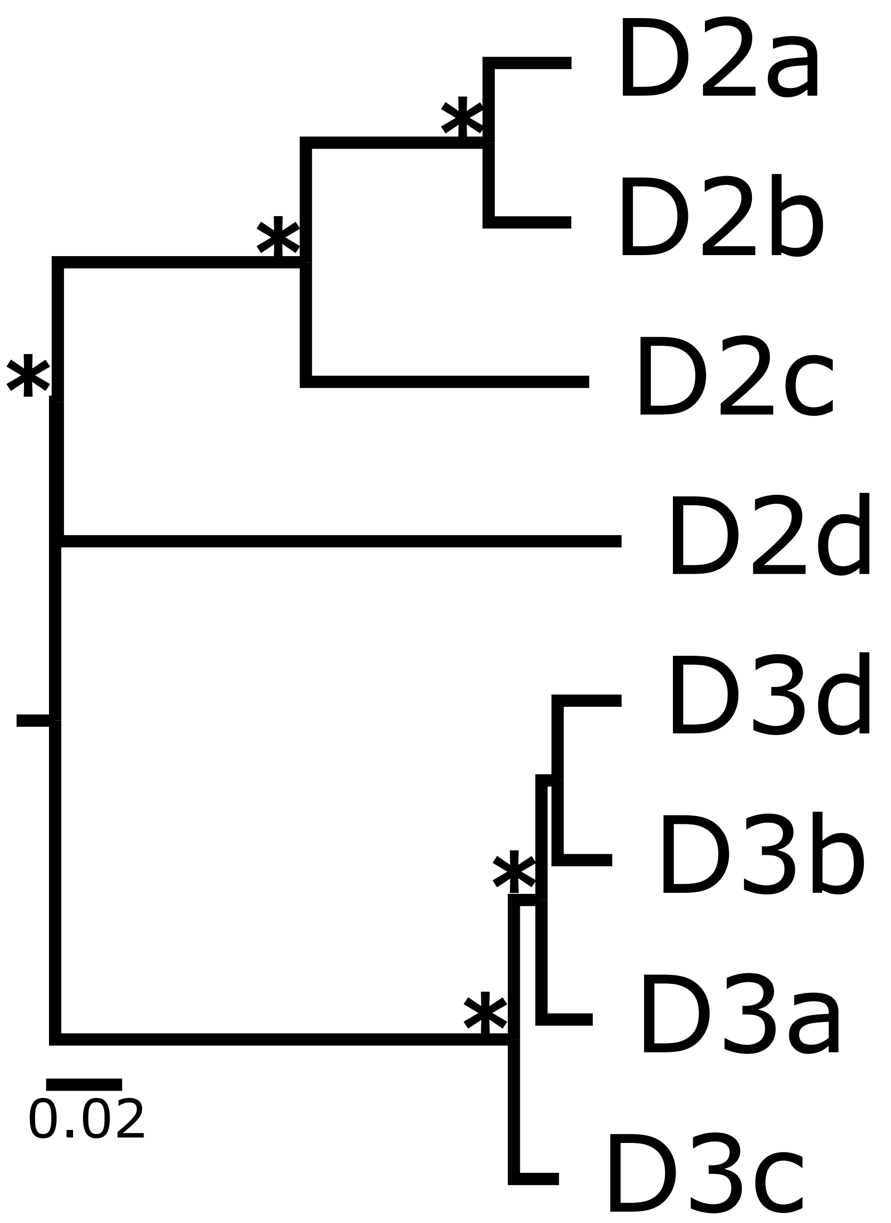

Supplementary Figure S3. The phylogenetic tree inferred from the maximum likelihood (ML) analysis based on the single nucleotide polymorphisms (SNP) dataset. Asterisks at the nodes indicate maximum likelihood bootstrap support of 99 or 100. The scale bar indicates substitutions per site.
