# Supplementary material for: High genomic differentiation and limited gene flow indicate recent cryptic speciation within the genus Laspinema (cyanobacteria)
Source: Front Microbiol. 2022 Sep 9;13:977454. doi: 10.3389/fmicb.2022.977454 (PMC9500459; doi:10.3389/fmicb.2022.977454)
Supplement: Supplementary file 1 [file Data_Sheet_1.ZIP › Supplementary Figure S4 rev.pdf]

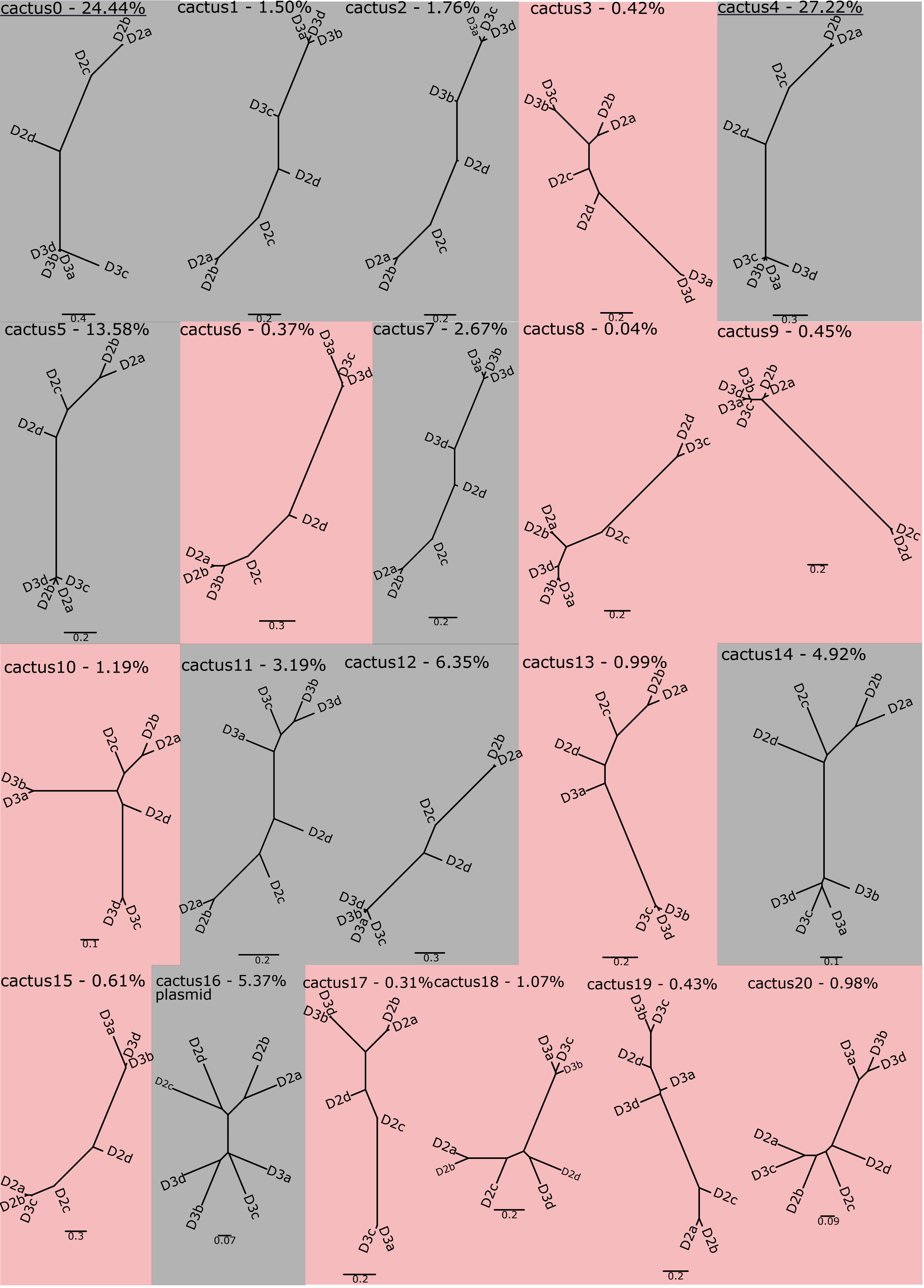

Supplementary Figure S4. All 21 local topologies (cacti) inferred by Saguaro over the genome with the percentage of the genome each cactus covered. Colors of the boxes correspond to the colors in Figure 1C. Cacti in dark grey boxes support the differentiation of the genome into D2 and D3 clades. Cacti in red boxes do not support it. Underlined cacti (cactus0 and cactus4) covered over 50% of the genome.
