# Supplementary material for: High genomic differentiation and limited gene flow indicate recent cryptic speciation within the genus Laspinema (cyanobacteria)
Source: Front Microbiol. 2022 Sep 9;13:977454. doi: 10.3389/fmicb.2022.977454 (PMC9500459; doi:10.3389/fmicb.2022.977454)
Supplement: Supplementary file 1 [file Data_Sheet_1.ZIP › Supplementary Figure S5.pdf]

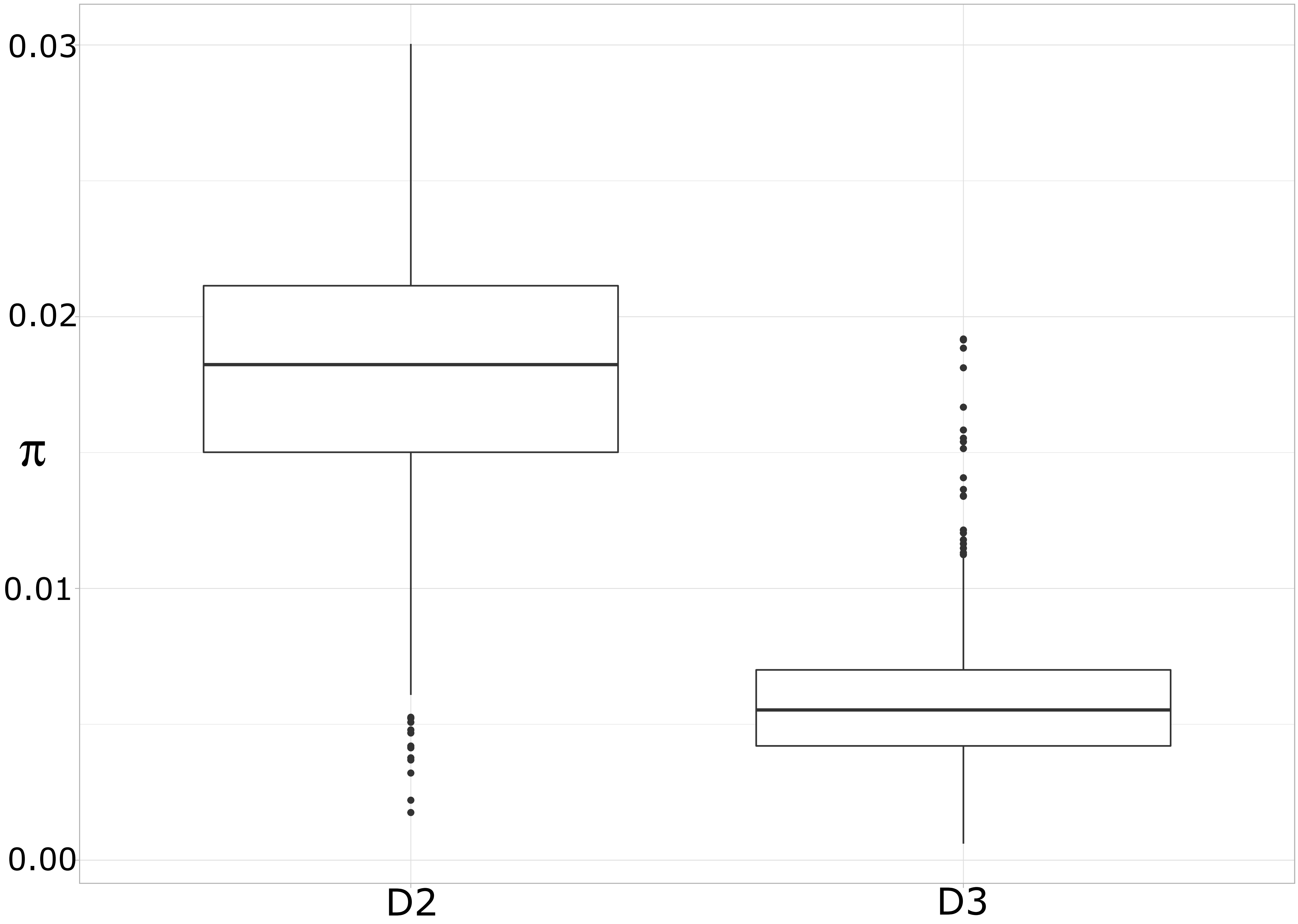

Supplementary Figure S5. Box plots representing the distribution of the genetic diversity (π) for D2 and D3 lineages
