# Supplementary material for: High genomic differentiation and limited gene flow indicate recent cryptic speciation within the genus Laspinema (cyanobacteria)
Source: Front Microbiol. 2022 Sep 9;13:977454. doi: 10.3389/fmicb.2022.977454 (PMC9500459; doi:10.3389/fmicb.2022.977454)
Supplement: Supplementary file 1 [file Data_Sheet_1.ZIP › Supplementary Figure S6.pdf]

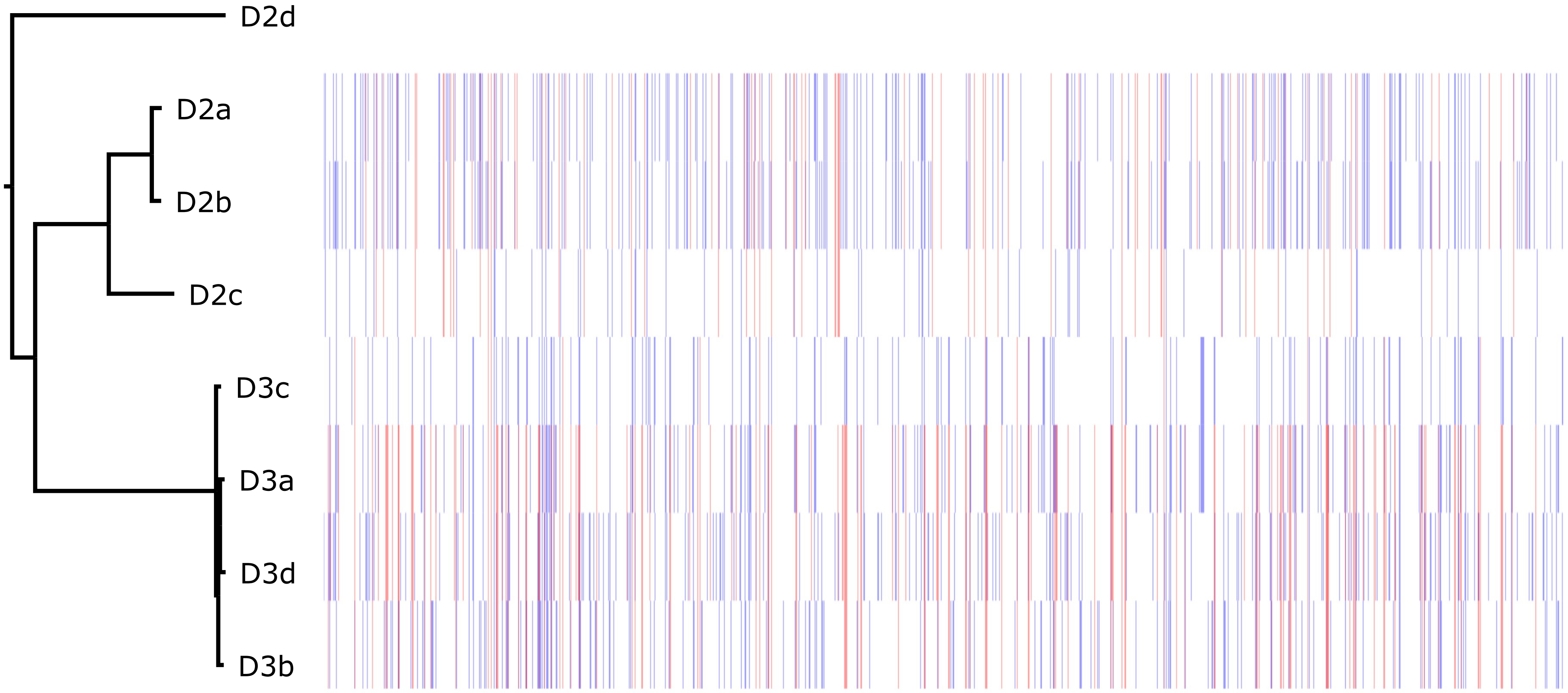

Supplementary Figure S6. Phylogenetic tree and recombination sites of eight Laspinema strains estimated by gubbins. The phylogenetic tree constructed via RAxML based on SNPs is shown on the left. Horizontal bars on the right indicate predicted recombination events for each Laspinema strain. The red color indicates recombination events present in multiple strains (more than one) and the blue color shows events predicted in a single strain.
